# A Genotype–Phenotype Analysis of Four Chinese Children Carrying Distinct Pathogenic Variants in the *CTCF* Gene

**DOI:** 10.3390/genes17090995

**Published:** 2026-08-24

**Authors:** Juan Du, Muhan Li, Aimin Liang, Mingyan Hei, Xiaotun Ren

**Affiliations:** 1 Children’s Health Care Center, Beijing Children’s Hospital, Capital Medical University, National Center for Children’s Health, Beijing 100045, China; 2008du_juan@163.com (J.D.); mh66mail@163.com (M.L.); 2 Neonatal Center, Beijing Children’s Hospital, Capital Medical University, National Center for Children’s Health, Beijing 100045, China; heimingyan@bch.com.cn; 3 Department of Neurology, Beijing Children’s Hospital, Capital Medical University, National Center for Children’s Health, Beijing 100045, China; rxt1997@126.com

**Keywords:** *CTCF* gene, autosomal dominant intellectual disability type 21, genotype–phenotype correlation, developmental delay, chylothorax, copy number variation, de novo variant

## Abstract

**Objective:** The objective of this study was to analyze the clinical phenotypes and genetic variant characteristics of four unrelated Chinese children carrying pathogenic variants in the *CTCF* gene and to explore the genotype–phenotype heterogeneity of autosomal dominant intellectual disability type 21 (MRD21, OMIM 615,502). **Methods:** Four pediatric patients admitted to Beijing Children’s Hospital, Capital Medical University, from 2020 to 2024 were enrolled in this retrospective study. All patients underwent medical history collection, physical examination, laboratory tests and high-throughput sequencing. Identified variants were verified in the probands and parents via Sanger sequencing or CNV-seq. **Results:** Four distinct heterozygous *CTCF* variants were identified: a missense variant c.1117C>T (p.His373Tyr); an 8.92 Mb microdeletion at 16q21-q22.1 (arr[GRCh37] 16q21q22.1(58,986,875–67,907,636)×1), encompassing the entire *CTCF* gene; a frameshift deletion c.615_618delGAAA (p.Lys206Profs*15); and an intragenic deletion of exons 8–10. Parental testing confirmed that all identified variants were of de novo origin. The missense variant and the frameshift deletion have been reported previously in ClinVar (VCV000521287.19 and VCV001308122.2), but the two deletions were not found in public databases. Three patients presented with global developmental delay consistent with MRD21, with variable additional features including autistic-like behavior and facial dysmorphism. Notably, two of these patients showed somatic overgrowth with height and weight above the 97th percentile, contrasting with the short stature classically emphasized in MRD21. The fourth patient was a preterm infant with neonatal chylothorax, cardiopulmonary failure, and multiple congenital cardiovascular malformations; developmental assessment at a corrected age of 11 months showed only mild lags. **Conclusions:** This study expands the spectrum of pathogenic *CTCF* variants in the Chinese population and underscores the marked phenotypic heterogeneity of CTCF-related disorders, ranging from benign developmental outcomes with complete catch-up to severe neonatal multisystem involvement. In neonates presenting with unexplained chylothorax and multisystem abnormalities, especially when accompanied by features suggestive of a neurodevelopmental syndrome, *CTCF* should be considered in the differential diagnosis. Given this heterogeneity, a broad genomic approach rather than targeted CTCF screening is recommended in patients with complex presentations.

## 1. Introduction

Autosomal dominant intellectual disability type 21 (MRD21) is a genetic disorder caused by pathogenic variants in the *CTCF* gene (CCCTC-binding factor; OMIM 604,167) [1]. Located at chromosome 16q22.1, *CTCF* encodes a widely expressed, multifunctional zinc finger protein that serves as a critical insulator-binding protein in vertebrates. In 2013, Gregor et al. first reported the association between *CTCF* variants and intellectual disability in humans [2]. CTCF recognizes and binds to over 30,000 specific genomic sites, participating in essential cellular processes by forming chromatin loops, regulating transcription and RNA splicing, maintaining imprinted gene expression patterns, and mediating epigenetic modifications [3,4,5]. As a key boundary factor for topologically associating domains (TADs), CTCF acts as an insulator to separate functionally distinct gene clusters. It mediates the interaction of promoters with distal regulatory elements, including enhancers and silencers, thereby influencing the activity and spatiotemporal specificity of gene expression [6,7]. Furthermore, the CTCF protein can activate or repress target gene transcription either by directly binding to promoters or by mediating the recruitment of other transcription factors [8,9,10].

CTCF dysfunction has been implicated in a broad spectrum of human diseases. While its role in malignant tumors has been extensively investigated, alterations at CTCF-binding sites, rather than in the *CTCF* gene itself, have been implicated in the pathogenesis of Huntington’s disease and autoimmune disorders [11,12]. Developmentally, the loss of CTCF-binding sites within the H19 differentially methylated region (DMR) leads to Beckwith–Wiedemann syndrome, a condition characterized by overgrowth and an increased predisposition to tumors [13,14]. Moreover, genome-wide association studies (GWASs) have demonstrated that single-nucleotide polymorphisms (SNPs) within the *CTCF* gene are significantly associated with human height [15,16]. The current literature indicates that diverse *CTCF* variations, including nonsense, frameshift, and missense variants, as well as genomic deletions, are linked to intellectual disability, microcephaly, and growth retardation [1]. Specifically, MRD21 is a rare genetic syndrome clinically defined by developmental delay, intellectual disability, short stature, and microcephaly. A subset of patients also presents with mild facial dysmorphism, such as a small mouth, prominent incisors, and a thin upper lip; structural cardiac anomalies, including atrial septal defects and patent ductus arteriosus; and autism spectrum behaviors [17,18].

This study performed clinical examinations and genetic analyses on four pediatric patients carrying variants in the *CTCF* gene. Three of them presented typical clinical manifestations of MRD21 with significant global developmental delay; some were additionally complicated by microcephaly, short stature, low birth weight, autism-like behaviors and other symptoms. One critically ill newborn exhibited a multisystem involvement phenotype. The clinical and genetic data of each patient are elaborated in detail below.

## 2. Subjects and Methods

### 2.1. Subiects

#### 2.1.1. Patient 1

Patient 1, a 3-year-and-11-month-old boy, presented for an evaluation of limited speech and poor social engagement. He began speaking intentionally at 22 months of age, initially using single and reduplicated syllables. Currently, he can formulate simple sentences primarily to express basic needs; however, his speech is dominated by echolalia and unintelligible vocalizations. Socially, he does not respond to his name, makes only fleeting eye contact, and follows simple commands only. He exhibits notable deficits in nonverbal communication, such as a lack of head nodding or shaking, and joint attention, including the absence of pointing, showing, or sharing objects. While he does not interact with peers, he is capable of interactive play with family members and rudimentary imaginative play. Although no prominent stereotypic behaviors were observed, his play patterns are highly restrictive and repetitive.

Birth and Developmental History: The patient was born full-term via vaginal delivery following a precipitous labor, with a birth weight of 3.4 kg. The perinatal period was unremarkable, with no history of hypoxia, resuscitation, or jaundice, and the pregnancy was uncomplicated. His developmental milestones were significantly delayed: he sat at 9 months, crawled at 13 months, stood with support at 15 months, and walked independently at 22 months. Currently, he is able to run but exhibits an uncoordinated gait and cannot stand on one foot. He also lacks bowel and bladder control and cannot dress himself. There is no family history of neurodevelopmental or language delays.

Past Medical History: At 1.5 years of age, he was diagnosed with global developmental delay at another institution. A brain magnetic resonance imaging (MRI) scan at that time reportedly revealed congenital brain developmental abnormality with right hippocampal encephalomalacia. Consequently, he underwent multidisciplinary rehabilitation for over a year, encompassing sensory integration, physical therapy (PT), occupational therapy (OT), cognitive therapy, and applied behavior analysis (ABA).

Physical Examination and Clinical Assessments: At presentation, both his height and weight were above the 97th percentile for his age. Mild facial dysmorphism was noted, including mild hypertelorism and a single transverse palmar crease. Behavioral observation confirmed poor eye contact and limited social reciprocity. Standardized assessments using the Gesell Developmental Diagnostic Schedule indicated severe global delays: Adaptive 34, Gross Motor 47, Fine Motor 28, Language 33, and Personal–Social 21. His Social Living Skills Assessment standard score was 7, indicating moderate impairment. Autism screening scales: The total score of the Clancy Autism Behavior Scale (CABS) was 20 points, including 1 item rated “never” and 7 items rated “often”; the Autism Behavior Checklist (ABC) score was 64 points. Both screening results indicated the presence of autism-like behaviors.

Laboratory and Imaging Findings: Hearing screening, thyroid function tests, liver and renal function panels, and a repeat brain MRI were all unremarkable. The metabolic screening of blood and urine revealed mildly elevated levels of 2-hydroxyisovaleric acid and 2-hydroxyisohexanoic acid.

Follow-up: During a telephone follow-up at 8 years and 6 months of age, the patient exhibited somatic overgrowth (height 145 cm, weight 53 kg, BMI 25.2; all >P97). Despite continuous multi-institutional rehabilitation since his initial presentation, no significant clinical improvement was noted; his intellectual disability and autism-like behaviors persist, and he has never attended school.

#### 2.1.2. Patient 2

Patient 2, a 10-month-old girl, presented for an evaluation of growth retardation and developmental delay. Growth faltering was initially noted at 6 months of age. Developmentally, she achieved supported sitting at 9 months but remained unable to sit independently by 10 months. Although she could crawl on all fours, she lacked age-appropriate fine motor and social milestones, such as exhibiting a pincer grasp or performing waving gestures.

Birth and Family History: The patient was born full-term via normal vaginal delivery, with a birth weight of 2.65 kg and a birth length of 48 cm. There was no perinatal history of hypoxia or asphyxia. Prenatal history was notable for a single umbilical artery. Prior to this presentation, she had not attended regular well-child physical examinations. The family history was unremarkable, with normal parental statures (father: 173 cm; mother: 160 cm).

Physical Examination: Anthropometric measurements revealed severe global growth restriction and microcephaly: height was 65 cm (<P3), weight was 6.5 kg (<P3), and head circumference was 41.4 cm (<P3). The anterior fontanelle was notably enlarged, measuring 4 × 4 cm, with two erupted teeth. Craniofacial dysmorphism included frontal bossing, hypertelorism, low-set ears, and a depressed nasal tip. Neurologically, she exhibited sluggish responsiveness, failed to respond to her name, and displayed stereotypic hand-flapping behaviors.

Clinical Assessments and Imaging: Standardized assessment using the Gesell Developmental Diagnostic Schedule (DQ) indicated significant global delays: Adaptive 69, Gross Motor 50, Fine Motor 64, Language 50, and Personal–Social 69. Hearing screening and echocardiography were both unremarkable. A non-contrast brain MRI revealed nodular T2 isointense lesions within the fourth ventricle, presumed benign but associated with secondary obstructive hydrocephalus, and widened extra-axial spaces in both temporal regions.

Follow-up: The patient has been receiving ongoing rehabilitation training at a local hospital. By 19 months of age, she could crawl but was unable to walk independently. At 22 months, her physical growth remained severely restricted (height 78.1 cm, <P3; weight 7.8 kg, <P3), and the anterior fontanelle had nearly closed (0.3 × 0.3 cm). Although she had achieved independent walking, her gait was unsteady. Her behavioral deficits persisted, characterized by a continued lack of response to her name and stereotypic hand-flapping.

#### 2.1.3. Patient 3

Patient 3, a 1-year-old girl, presented for an evaluation of developmental delay. At the time of presentation, she was nonverbal, exhibited delayed responses to instructions, and showed overall sluggish responsiveness. However, her gross motor development was age-appropriate, she maintained normal eye contact, and she displayed no obvious stereotypic or repetitive behaviors.

Birth and Family History: The patient was born full-term at 39 weeks and 4 days of gestation via cesarean section, with a birth weight of 3.0 kg and a birth length of 49 cm. Her past medical history was unremarkable, and family history was unremarkable for genetic or neurodevelopmental disorders.

Physical Examination: Anthropometric measurements indicated rapid somatic growth, with a height of 81.5 cm (>P97) and a weight of 11 kg (P90–P97). Significant craniofacial dysmorphism was absent, although left-sided strabismus was observed. Behavioral observation during the examination confirmed adequate eye contact and social engagement.

Clinical Assessments and Imaging: Standardized evaluation using the Gesell Developmental Diagnostic Schedule (DQ) indicated mild language and personal–social delays but relatively preserved motor skills: Adaptive 79, Gross Motor 96, Fine Motor 86, Language 67, and Personal–Social 71. Hearing screening and electroencephalography (EEG) were completely normal. A brain MRI revealed a cystic lesion in the right temporal pole.

Follow-up: During a telephone follow-up at 4 years and 8 months of age, her physical growth remained accelerated (height 122 cm, >P97; weight 21 kg, P90–P97). Following her initial presentation, she underwent six months of targeted rehabilitation therapy at a local hospital, focusing on language, social, and motor skills. She began speaking at approximately 18 months of age, followed by a rapid period of language acquisition. At follow-up, her global development was commensurate with that of age-matched peers.

#### 2.1.4. Patient 4

Patient 4, a female infant, presented at 5 days of life for an evaluation of persistent respiratory distress since birth.

Birth and Maternal History: The infant was born prematurely at 33 weeks and 3 days of gestation via cesarean section indicated by the prenatal ultrasound detection of fetal pleural effusion. Birth weight was 1.74 kg, and birth length was 46 cm. Apgar scores were 8, 9, and 9 at 1, 5, and 10 min, respectively. The pregnancy was complicated by gestational diabetes. The mother had previously delivered a healthy 5-year-old boy and had one spontaneous abortion at 2 months of gestation attributed to progesterone deficiency. The parents are healthy and non-consanguineous.

Neonatal Intensive Care Unit (NICU) Course: The neonate presented with tachypnea and labored breathing immediately after birth, accompanied by hypoglycemia with a capillary glucose of 1.93 mmol/L. Hypovolemic shock developed on postnatal day 3 at an outside hospital. Chest CT showed bilateral pneumonia, pleural effusion, and right lung atelectasis; acellular chylous fluid was drained from the right pleural cavity. Due to persistent clinical deterioration, the infant was transferred to our NICU on postnatal day 5. The clinical course was characterized by severe multisystem involvement, including chylothorax, preterm encephalopathy, respiratory failure requiring high-frequency oscillatory ventilation, heart failure, neonatal pneumonia, pericardial effusion, feeding intolerance, neonatal hypoglycemia, congenital heart defects (patent ductus arteriosus [PDA], patent foramen ovale [PFO]), pulmonary hypertension, neonatal jaundice, anemia necessitating blood product transfusion, and recurrent severe gastro-esophageal reflux that precluded enteral feeding. The infant was discharged with a nasojejunal tube after 61 days.

Physical Examination on Admission: Anthropometric measurements adjusted for corrected gestational age were as follows: length 46 cm (P50–90), weight 1.56 kg (P10–50), and head circumference 31 cm (P50–90). Clinical examination revealed mild jaundice, diminished breath sounds over the right lung field, single transverse palmar creases, and low-set ears. Neurologically, she exhibited generalized hypotonia and incomplete primitive neonatal reflexes.

Laboratory and Imaging Findings: Repeat chest CT confirmed bilateral pneumonia, pleural effusions, right lower lobe atelectasis, and pericardial effusion. Echocardiography revealed a PFO/atrial septal defect (ASD), a PDA, minimal pericardial effusion, and mildly reduced left ventricular ejection fraction (LVEF 53.8%). A cranial ultrasound showed mildly increased periventricular white matter echogenicity. Non-contrast head CT revealed mildly decreased white matter density in the bilateral frontoparietal lobes and left lateral ventriculomegaly. Renal ultrasonography was unremarkable. Electroencephalogram was mildly abnormal, characterized by excessive tracé alternant patterns during quiet sleep. Bronchoscopy revealed congenital laryngomalacia. Hearing screening passed for auditory brainstem response bilaterally; otoacoustic emissions passed in the right ear but referred in the left. Laboratory test revealed hypocalcemia with a total serum calcium of 1.6 mmol/L, hyponatremia with a serum sodium of 127.7 mmol/L, and hyperbilirubinemia with a total bilirubin of 164.41 μmol/L, direct bilirubin of 22 μmol/L, and total bile acids of 73.55 μmol/L. Thyroid function was normal.

Follow-up: The nasojejunal tube was accidentally dislodged approximately 2 months after discharge, after which the patient transitioned to full oral feeding. At 6 months and 9 days of age (corrected age 4 months and 23 days), somatic growth parameters were height 63.7 cm (P25–50), weight 6.09 kg (P3–10), and head circumference 38 cm (<P3). Hearing, vision, and metabolic screenings (blood/urine) were normal. Head CT showed mildly widened lateral ventricles, and echocardiography revealed a persistent PFO with a left-to-right shunt. At 13 months and 12 days of age (corrected age 11 months and 26 days), growth had improved to height 76.8 cm (P75–90), weight 10.71 kg (P75–90), and head circumference 42.4 cm (P10–25). Cranial ultrasound demonstrated bilateral lateral ventriculomegaly with a left lateral ventricular index of 1.60 cm and a right lateral ventricular index of 1.59 cm. Pulmonary ultrasound revealed mild alveolar–interstitial syndrome bilaterally. Developmental assessment using the Griffiths Developmental Scales-Chinese (GDS-C) showed mild global lags, with subscales mapping closely to the 10.5- to 11-month-old level: Gross Motor 17.50% (10.5 months), Personal–Social Skills 20.00% (10.5 months), Hearing and Language 17.50% (10.0 months), Hand–Eye Coordination 30.00% (11.0 months), and Visual Performance 20.00% (10.5 months). The main clinical characteristics of the four patients are listed in Table 1.

### 2.2. Methods

#### 2.2.1. Sample Collection and DNA Extraction

Peripheral blood samples were collected from the probands (4 mL) and their respective parents (2 mL each) in EDTA-anticoagulated tubes. Genomic DNA was extracted using the FlexiGene DNA Kit (Qiagen, Hilden, Germany) according to the manufacturer’s protocol. DNA concentration and purity were assessed using a NanoDrop 2000 spectrophotometer (Thermo Fisher Scientific, Waltham, MA, USA), followed by standard quality control procedures.

#### 2.2.2. Genetic Testing

##### Whole-Exome Sequencing (WES)

Genomic DNA was sheared into 100–500 bp fragments using a Covaris ultrasonicator (Covaris, Woburn, MA, USA). Libraries were constructed using a target sequence enrichment kit (Agilent Technologies, Santa Clara, CA, USA), and targeted capture probes were employed to enrich the entire exome (all protein-coding regions of the genome) along with flanking intronic sequences (±10 bp). The enriched target fragments were amplified by a polymerase chain reaction (PCR), and the products underwent purification and rigorous quality assessment. Based on the DNA concentration of the captured libraries and the required sequencing depth, paired-end sequencing was performed on an Illumina next-generation sequencing (NGS) platform (Illumina, San Diego, CA, USA).

Bioinformatic analysis was performed using a customized pipeline. Raw sequencing data were converted into standard FASTQ format using CASAVA software (version 1.8.2). Sequence alignment, variant calling, and annotation were conducted following established protocols. Variant interpretation and pathogenicity classification were performed in strict accordance with the American College of Medical Genetics and Genomics (ACMG) guidelines, with the aim of identifying pathogenic or likely pathogenic variants that correlated with the patients’ clinical phenotypes. Candidate variants identified via WES that were highly consistent with the clinical presentations were validated using Sanger sequencing (Table 2). Parental segregation analysis was additionally performed using the parents’ genomic DNA to confirm the origin and inheritance pattern of the variants.

##### MLPA Assay

Multiplex ligation-dependent probe amplification (MLPA) was performed using the SALSA MLPA Probemixes P070 (subtelomeres) and P106 (X-linked ID) (MRC-Holland, Amsterdam, The Netherlands). The experimental procedures, performed in strict accordance with the manufacturer’s protocols, encompassed sample quality control, DNA denaturation, probe hybridization, ligation, and PCR amplification. The amplified fragments were subsequently separated, and their peak heights and areas were quantified using an ABI 3130 Genetic Analyzer (Applied Biosystems, Foster City, CA, USA). Data interpretation and quantitative analysis were performed using Coffalyser.Net software (MRC-Holland, v.220513.1739).

##### Cytogenomic Array Analysis

Chromosomal microarray analysis (CMA) was performed using the CytoOneArray platform to identify genomic imbalances (microdeletions and microduplications) across 326 disease-associated regions and 41 subtelomeric regions. Following the co-hybridization of patient and reference DNA samples, microarray images were processed using CytoCloud^®^ analysis software. Copy number variations (CNVs) larger than 100 kb or encompassing more than three consecutive valid probes were filtered for further assessment. The clinical significance of the detected CNVs was systematically evaluated in accordance with ACMG guidelines, with reference to publicly available databases including ClinVar, DECIPHER, OMIM, GeneReviews, Orphanet, and the Database of Genomic Variants (DGV).

##### Copy Number Variation Sequencing

Genomic DNA was extracted from the peripheral blood samples using the FlexiGene DNA Kit (Qiagen, Germany). For library construction, the genomic DNA was subjected to ultrasonic shearing to generate 150–300 bp fragments, followed by end-repair, adapter ligation, and PCR amplification. The quality of the constructed libraries was subsequently assessed using an Agilent 2100 Bioanalyzer (Agilent Technologies, Santa Clara, CA, USA). The qualified libraries were sequenced on an Illumina NovaSeq 6000 platform (Illumina, San Diego, CA, USA) using a paired-end sequencing strategy. Following quality control filtering, the raw sequencing reads were aligned to the human reference genome (GRCh37/hg19) using the BWA-MEM algorithm and SAMtools. Copy number variation (CNV) calling was performed using CNVkit, while variant annotation and clinical phenotype matching were performed with reference to publicly available databases, including UCSC, DGV, OMIM, and the Human Phenotype Ontology (HPO).

## 3. Results

### 3.1. High-Throughput Sequencing and Sanger Sequencing Validation

Exon numbering throughout this paper follows the RefSeq transcript NM_006565.4 as annotated by the original diagnostic laboratory; readers should note that alternative exon numbering conventions have been used in earlier reports, and all variants are therefore also given as c. coordinates.

**Figure 1:**
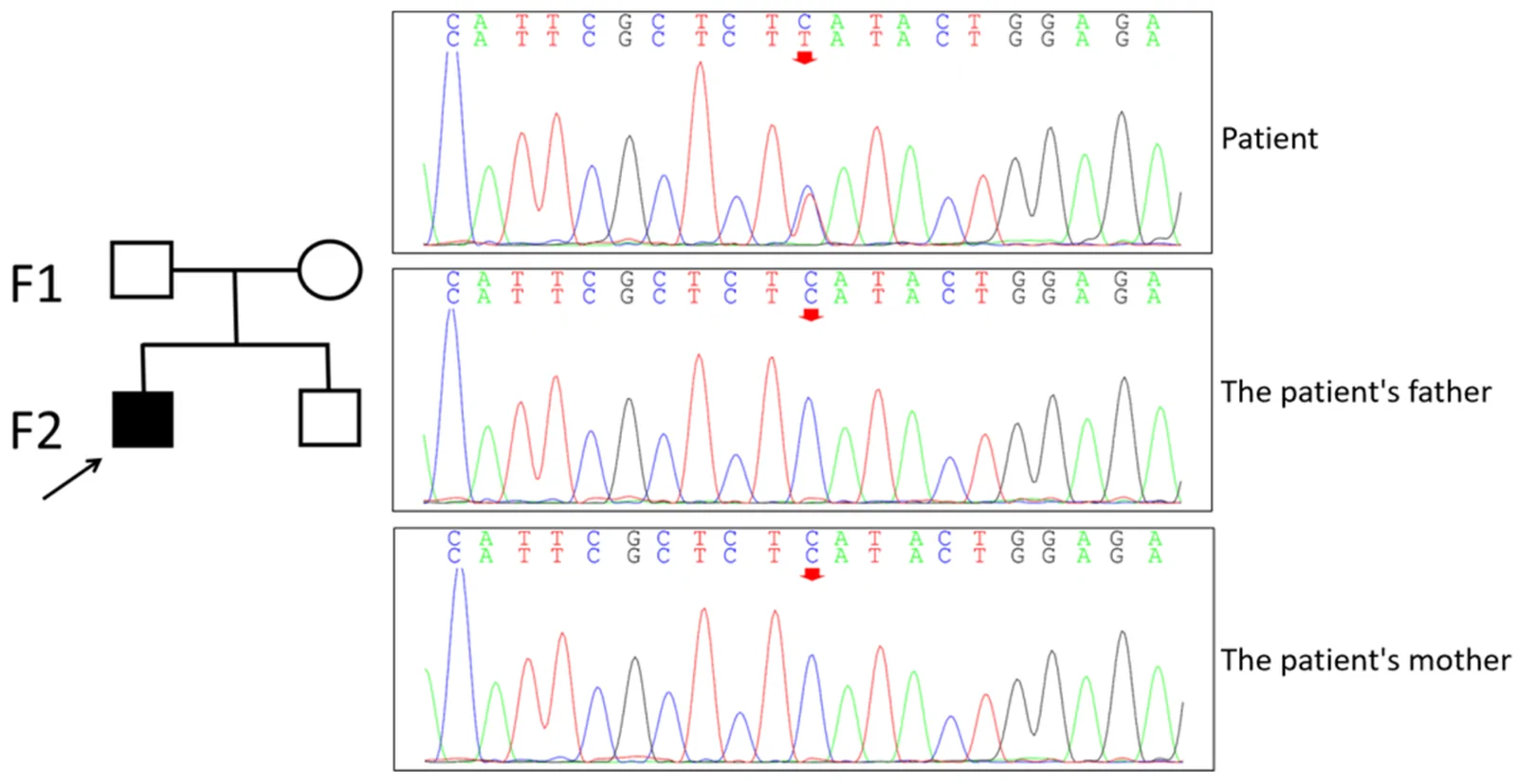
Family tree for Patient 1 and Sanger sequencing chromatograms for patient and parents. (Note: Red arrow points to c.1117C>T variant site. Black arrow indicates the patient).

For Patient 1, whole-exome sequencing identified a heterozygous missense variant in exon 6 of the *CTCF* gene. The variant is c.1117C>T in the cDNA sequence and p.His373Tyr in the protein sequence (Figure 1). Chromosomal microarray analysis and MLPA with probemixes P070 and P106 were negative. The variant is recorded in ClinVar under accession VCV001308122.2.

For Patient 2, the CNV-seq of the proband detected a pathogenic copy number loss of approximately 8.92 Mb at 16q21-q22.1. The deletion is designated as NC_000016.9:g.58986875_67907636del. No other clinically relevant imbalance was identified. The sex chromosome complement was 46,XX.

For Patient 3, whole-exome sequencing identified a heterozygous 4 bp deletion in exon 3 of the *CTCF* gene. The variant is c.615_618delGAAA in the cDNA sequence and p.Lys206Profs*15 in the protein sequence, introducing a frameshift and a premature stop codon. The variant is recorded in ClinVar under accession VCV000521287.19 and has been reported previously in individuals with a CTCF-related neurodevelopmental disorder [17,18] (PMIDs 30893510 and 31239556).

For Patient 4, a copy number analysis of the whole-exome sequencing data detected a heterozygous multi-exon deletion encompassing exons 8 through 10 of the *CTCF* gene. The precise genomic breakpoints could not be resolved from exome coverage data. The deletion is reported using HGVS notation for uncertain breakpoints.

**Figure 2:**
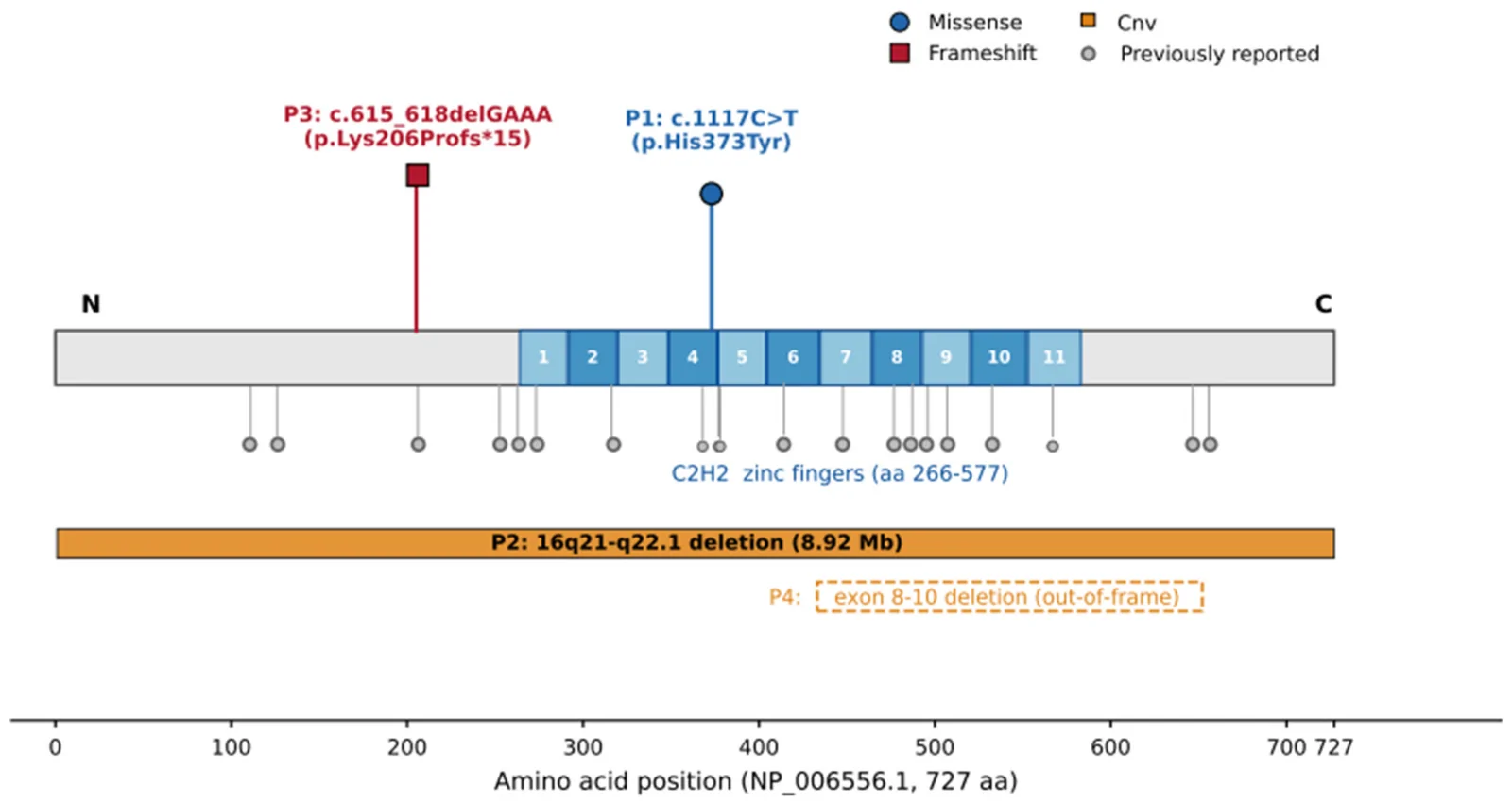
A schematic diagram of the CTCF protein (NP_006556, 727 amino acids) and the distribution of the variants identified in this study. The 11 tandem arrays of C2H2 zinc fingers are represented in blue, with the domain boundaries referring to the database UniProt (P49711). The identified variants in this series are color-coded according to their protein consequences; previously reported CTCF variants associated with neurodevelopmental phenotypes are displayed in gray below. The horizontal bars represent copy number deletions.

Sanger sequencing and quantitative validation of the parent–offspring trios confirmed that none of the respective parents carried these alterations. All four variants were therefore confirmed to be de novo. The variants appeared to distribute in multiple locations across the whole gene (Figure 2).

### 3.2. Pathogenicity Analysis of Identified Variants

The four variants were classified according to the American College of Medical Genetics and Genomics guidelines (Table 3).

For Patient 1, the heterozygous missense variant c.1117C>T (p.His373Tyr) was identified in exon 6 of the *CTCF* gene. A search across adult population databases revealed that this variant is absent in ExAC, ESP6500, and the 1000 Genomes Project (including the CHB and CHS subpopulations). Its allele frequency in the Kangso Health database is also 0.0, confirming its rarity in the general population and fulfilling the moderate pathogenicity evidence (PM2). Three in silico predictive algorithms—SIFT (Deleterious), PolyPhen-2 (Probably damaging), and MutationTaster (Disease-causing)—consistently predicted a deleterious effect on the gene product’s function, satisfying the supportive evidence (PP3). The variant lies within the tandem array of 11 C2H2 zinc fingers that occupies the central portion of CTCF (approximately amino acids 266–577; UniProt P49711), a region in which pathogenic missense variation is concentrated: all 20 missense variants in the cohort of Konrad et al. were located in exons encoding one of the 11 zinc fingers [18], and a systematic catalog of CTCF variants has shown an enrichment in disease-associated missense changes within the zinc finger array together with the depletion of population missense variation in the same region [24]. His373 is itself one of the two zinc-ligating histidines of the fourth zinc finger [25], and the zinc finger residues of CTCF are the principal determinants of its DNA-binding behavior [25,26]. PM1 was therefore applied. In addition, His373 is one of the seven residues recurrently affected in the same cohort, having been substituted once by aspartate (p.His373Asp) and once by proline (p.His373Pro) in individuals with a CTCF-related neurodevelopmental disorder [18]; PM5 is therefore satisfied. Pedigree analysis confirmed that the phenotypically normal parents do not carry this variant, verifying it as a de novo variant (PS2). Based on the combined evidence (PS2 + PM1 + PM5 + PM2_Supporting + PP3), applied according to the ACMG/AMP guideline [21], this missense variant is classified as “likely pathogenic”.

For Patient 2, the CNV-seq analysis revealed a microdeletion at 16q21-q22.1, indicating that the patient carries a large chromosomal deletion of approximately 8.92 Mb. The molecular localization is seq[GRCh37/hg19] 16q21-q22.1 (58,986,875–67,907,636)×1. This pathogenic CNV completely encompasses the entire *CTCF* gene. *CTCF* is a well-established haploinsufficient gene. The deletion arose de novo in the proband. According to the ACMG/ClinGen technical standard for constitutional copy number variants, the deletion was classified as pathogenic. The complete annotation of all genes within the deleted interval is provided in Supplementary Table S1.

For Patient 3, the heterozygous frameshift deletion c.615_618delGAAA (p.Lys206Profs*15) met the following evidence criteria. This variant is a classic loss-of-function variant introducing a premature termination codon. It is located far upstream of the penultimate exon boundary, a context in which nonsense-mediated mRNA decay is expected. The variant is absent from population databases including gnomAD v4.1.0. It was confirmed to be de novo. According to ACMG criteria, the pieces of evidence PVS1, PS2, and PM2_Supporting were applied. The variant was classified as pathogenic.

For Patient 4, the heterozygous intragenic deletion of exons 8 through 10 was an in-frame deletion. The reading frame consequence was assessed with the LOVD exonic deletion reading frame checker (CTCF, NM_006565.3, exons 8–10; https://databases.lovd.nl/shared/scripts/readingFrameChecker.php, accessed on 6 August 2026; Figure S1). The deleted region encodes the carboxy-terminal part of zinc finger 7 and the entire zinc fingers 8 through 11, a region critical to DNA-binding function as established by previous structural studies. Because the deletion preserves the reading frame, the PVS1 evidence was applied at strong rather than very strong weight. The deletion was confirmed to be de novo and is absent from population databases. According to ACMG criteria, the pieces of evidence PVS1_Strong, PS2, and PM2_Supporting were applied. The variant was classified as pathogenic. Because the deletion was called from exome coverage data, the precise genomic breakpoints could not be resolved; the deletion is therefore reported using HGVS notation for uncertain breakpoints, and the RNA-level confirmation of the predicted transcript consequence was not performed.

## 4. Discussion

The CTCF protein is widely expressed in vertebrates and predominantly localized in the cell nucleus. It belongs to the zinc finger protein family and comprises 727 amino acids, with three functional domains: the N-terminal domain, the central 11 zinc finger domains, and the C-terminal intrinsically disordered regions [25,26]. The N-terminal domain interacts with the cohesin complex to orchestrate three-dimensional chromatin architecture via loop extrusion [27]. The 11 zinc finger domains are responsible for the specific recognition and binding of target DNA sequences, with zinc fingers 3 to 7 constituting the core DNA-binding domain that governs the selection of genomic binding sites [28].

In Patient 1, the c.1117C>T (p.His373Tyr) missense variant affects zinc finger 4. His373 is one of the two zinc-ligating histidines of this finger [25], and crystallographic analyses show that zinc finger 4 contributes base-specific contacts to module 3 of the CTCF-binding site through Glu362, Lys365, and Arg368 [25,28]. Impaired zinc coordination would be expected to compromise the fold of this finger and to reduce binding preferentially at genomic sites that depend on it, rather than to abolish CTCF binding globally. This model is consistent with the observation that His373 is a recurrently mutated residue in CTCF-related disorder [18] and with the relatively selective phenotype of our patient, but it remains a structural inference that has not been tested functionally. In Patient 3, the c.615_618delGAAA (p.Lys206Profs*15) frameshift mutation in exon 3 introduces a premature stop codon, predicted to yield a truncated protein lacking all downstream zinc finger domains or to trigger nonsense-mediated mRNA decay. The truncation occurs upstream of the YxF/CES motif (amino acids 222–231, key residues Tyr226 and Phe228) through which CTCF engages the cohesin subcomplex [27]; any transcript escaping nonsense-mediated decay would lack both cohesin-anchoring and DNA-binding capacity. Patient 4 carried a heterozygous in-frame deletion of exons 8 to 10, predicted to remove zinc fingers 8 to 11 and the carboxy-terminal part of zinc finger 7 from an otherwise intact protein. The deleted segment includes the region that mediates cross-strand contacts for binding to the 5′ upstream motif of high-affinity CTCF sites [25,28]. This allele is thus expected to be expressed, and a dominant-negative contribution from a DNA-binding-deficient product cannot be excluded. By contrast, Patient 2 harbored a large heterozygous deletion of 8.92 Mb at 16q21-q22.1 that completely encompasses the entire *CTCF* gene.

**Figure 3:**
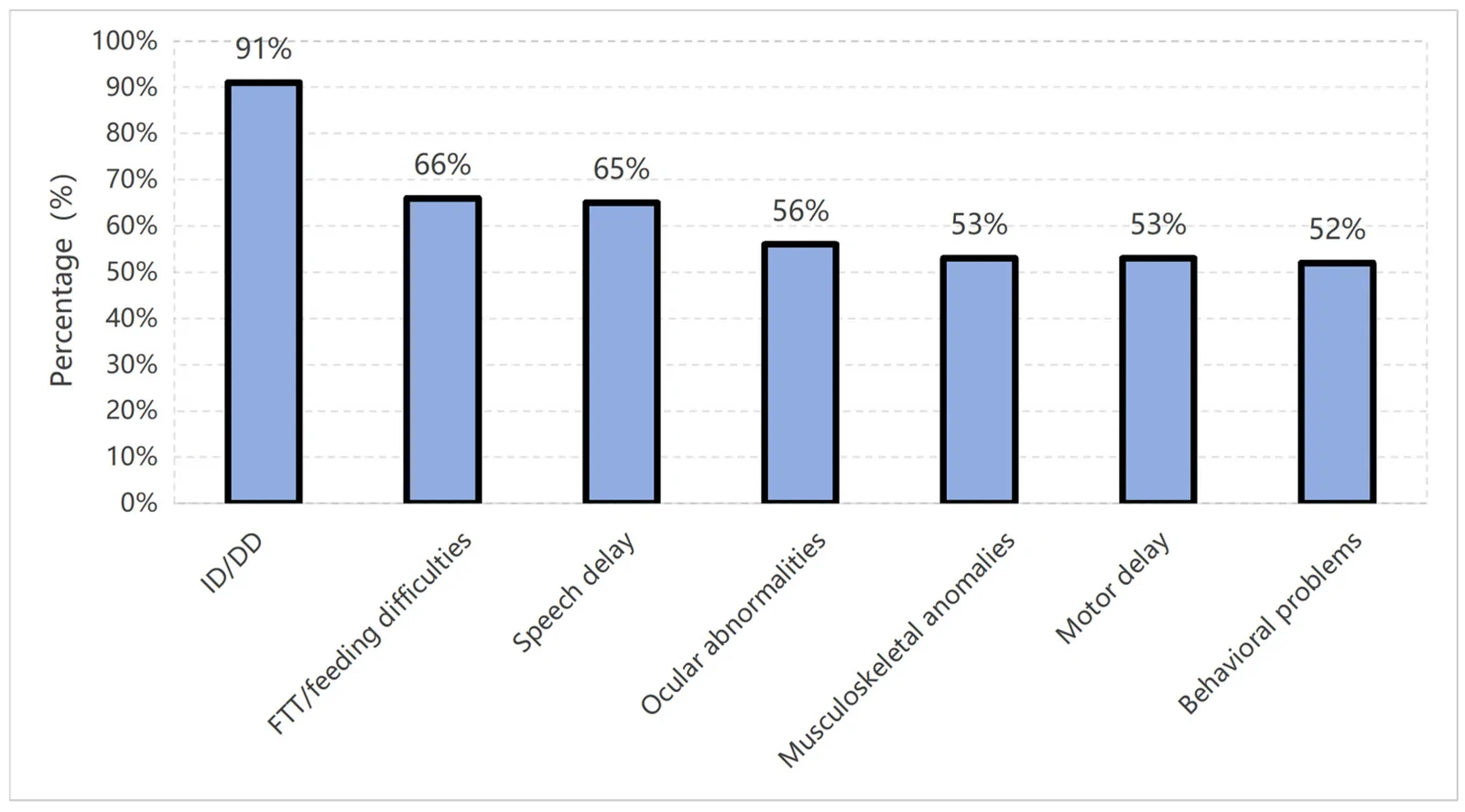
The frequency of the cardinal (more than 50%) clinical features of CTCF-related disorder across all reported subjects (*n* = 102). Abbreviations: ID, intellectual disability; DD, developmental delay; FTT, failure to thrive.

In 2013, Gregor et al. first reported the association between *CTCF* mutations and varying degrees of intellectual disability, microcephaly, and growth retardation [2]. A search of PubMed, CNKI, and Wanfang using CTCF as a keyword identified 107 reported cases of *CTCF* gene mutations associated with intellectual disability/developmental delay, speech delay, motor delay, feeding difficulties/failure to thrive, ocular abnormalities, musculoskeletal anomalies and behavioral problems (Figure 3), of which 86 were de novo mutations [29]. The phenotypes observed in the four patients in the present study are consistent with previously reported MRD21 cases. For example, patients carrying c.1186dupA, c.688delG, c.773_776del, c.804_805delTG, or c.782-2A>G presented with single transverse palmar creases or foot deformities; patients with c.688delG, c.773_776del, c.804_805delTG, c.782-2A>G, c.782-1G>T, or c.329dupT exhibited facial dysmorphism including hypertelorism; patients with c.1699C>T or c.1054_1055insA exhibited autistic behaviors; and patients with de novo mutations c.848G>A or c.1024C>T exhibited both autistic behaviors and developmental delay [17,18]. The patients in our cohort presented with similar features, including single transverse palmar creases, hypertelorism, autism spectrum behaviors, and developmental delay [17,18]. Notably, the missense variant affecting His373 in Patient 1 recurs at a residue previously reported by Konrad et al., who identified substitutions at this codon to aspartate and to proline in individuals with CTCF-related disorder [18]. The frameshift deletion c.615_618delGAAA in Patient 3 has also been reported previously in Chinese patients and in the Konrad cohort [17,18]. Among the four variants described here, two have been recorded in ClinVar, namely c.1117C>T (VCV001308122.2) and c.615_618delGAAA (VCV000521287.19); the 8.92 Mb 16q21-q22.1 deletion and the intragenic deletion of exons 8 to 10 were not retrieved from ClinVar, DECIPHER, dbVar, or PubMed at the time of our search. Tan et al. reported an adjacent in-frame deletion, c.1838_1852del (p.Glu613_Pro617del), in a Chinese patient with intellectual disability, and demonstrated by RNA sequencing that the mutant transcript was expressed rather than degraded, supporting the possibility that in-frame deletions in this region may exert their effect through a dominant-negative mechanism [28].

Currently, there is a substantial body of literature on the association between the CTCF gene and disease, but research on the specific pathogenic mechanisms of CTCF is relatively scarce. Gregor et al. investigated genomic variations in four patients with mild intellectual disability, autistic behaviors, short stature, microcephaly, cleft palate, and congenital heart defects. They found and confirmed that the c.375dupT/c.1186dupA mutations lead to reduced CTCF protein expression, indicating that CTCF haploinsufficiency can cause intellectual disability, while c.1699C>T may impair CTCF’s DNA-binding affinity and specificity through dominant-negative effects and haploinsufficiency, thereby affecting gene function [2]. Konrad et al. reported 39 individuals carrying CTCF aberrations, comprising 20 different missense variants, six frameshifting variants including a deletion of exon 8, two nonsense variants, two splice acceptor variants, and two large 16q22.1 deletions; among these, c.615_618del and c.1357+2201_1518+520del are relevant to the present series. They concluded that loss-of-function variants and haploinsufficiency underlie CTCF-associated neurodevelopmental disorders [18]. Hori et al. studied two patients with growth failure, developmental delay, and microcephaly, finding that both patients harbored deletion mutations in the CTCF gene, one with a 16q22.1 deletion at 67.1 to 68.2 Mb and the other with a 16q22.1 deletion at 67.3 to 68.9 Mb. Subsequent DNA methylation analysis revealed that DNA methylation at CTCF-binding sites was disrupted, which may represent the pathogenesis of CTCF syndrome [20].

Long-term follow-up data from previous cohorts have demonstrated that some children with only mild developmental delay exhibit favorable long-term outcomes, with cognitive development fully catching up to that of age-matched normal peers [18]. Case 3 in the present study is consistent with this benign phenotypic subgroup. This observation supports the notion that the phenotypic spectrum of CTCF-related disorder includes individuals with favorable developmental trajectories and that early intervention may contribute to positive outcomes. However, the variability in outcomes across patients with different variant types highlights the need for individualized prognostic counseling and long-term follow-up.

A small number of previous studies have reported neonates diagnosed with CTCF pathogenic variants via genetic testing due to congenital malformations and feeding difficulties in the neonatal period [30]. To our knowledge, no previous report has described a *CTCF* variant accompanied by neonatal chylothorax, cardiopulmonary failure and pulmonary hypertension. This combination may therefore extend the reported clinical spectrum of CTCF-related disorders. Several caveats must nevertheless be emphasized. First, the observation rests on a single neonate. Second, congenital chylothorax is etiologically heterogeneous: in a systematic analysis of published neonatal cases, chromosomal aberrations were identified in approximately 18%, most frequently Down, Noonan and Turner syndromes [31], and Patient 4 was additionally born at 33 weeks and 3 days of gestation, so prematurity-related contributions to her cardiorespiratory course cannot be separated from any genetic effect. Third, a coincidental, unrelated cause of the chylothorax cannot be excluded. It is relevant, however, that the deletion in this patient is confined to *CTCF*, so unlike a contiguous gene deletion, no neighboring gene can be invoked as an alternative explanation. Independent cases will be required before chylothorax can be regarded as part of the phenotype of CTCF-related disorder. Nonetheless, for neonates presenting with unexplained chylothorax accompanied by multisystem abnormalities and features suggestive of a neurodevelopmental syndrome, CTCF should be considered in the differential diagnosis, and a broad genomic approach is preferable to targeted gene panels.

The 8.92 Mb deletion in Patient 2 contains 87 annotated loci, 57 of which are protein-coding. Interstitial deletions of this segment are recognized as chromosome 16q22 deletion syndrome (OMIM #614,541) [19], and Hori et al. proposed that microdeletions encompassing CTCF constitute a CTCF deletion syndrome characterized by hypermethylation at CTCF-binding sites [20]. CTCF is the only gene in the interval with an established autosomal dominant haploinsufficiency-mediated neurodevelopmental phenotype and is likely the principal contributor to the developmental delay, microcephaly, and growth restriction in this patient. Several codeleted genes deserve mention. CBFB (MIM 121360) is the gene underlying cleidocranial dysplasia type 2 (CLCD2; MIM 620099), an autosomal dominant skeletal disorder caused by heterozygous loss-of-function variants [32]. The core features of CLCD2 include delayed fontanel closure, prominent frontal and parietal bones, delayed ossification, and dental anomalies [32]. An earlier patient with a 1.2 Mb 16q22 deletion encompassing CBFB had a large fontanel with delayed skull mineralization and multiple Wormian bones, suggesting that the haploinsufficiency of CBFB may contribute to the large fontanel in 16q22 deletion syndrome [19]. These features overlap with the enlarged anterior fontanelle (4 × 4 cm) and frontal bossing in our patient, so CBFB haploinsufficiency may contribute to the craniofacial phenotype. HSF4 (MIM 602438) is associated with autosomal dominant cataract (MIM 116800); the reported pathogenic changes are missense substitutions rather than whole-gene losses, so the consequence of hemizygosity is uncertain, but ophthalmological surveillance is advisable [19]. HSD11B2, CARMIL2, TK2, and CDH11 are associated with autosomal recessive conditions: apparent mineralocorticoid excess (MIM 218030), immunodeficiency 58 (MIM 618131), mitochondrial DNA depletion syndrome 2 (MIM 609560/617069), and Elsahy–Waters syndrome (MIM 211380). Hemizygosity would confer carrier status, but the unmasking of a recessive allele on the retained chromosome cannot be excluded, and the monitoring of blood pressure and serum potassium is reasonable given the HSD11B2 deletion. CDH5 (MIM 601120), encoding VE-cadherin, is also deleted; OMIM lists no Mendelian phenotype for CDH5 haploinsufficiency. ACD (MIM 609377) is associated with autosomal dominant dyskeratosis congenita 6 (MIM 616553), but the dominant mechanism is a specific loss of telomerase recruitment rather than haploinsufficiency [33], and ClinGen has curated no haploinsufficiency evidence for this gene. NOL3 is associated with familial cortical myoclonus 1 (MIM 614937), reported in a single family with a missense allele, and Nol3-null mice show no overt motor phenotype [34], arguing against haploinsufficiency. BEAN1 is associated with spinocerebellar ataxia 31 (MIM 117210), caused by a pentanucleotide repeat insertion acting through RNA gain of function rather than the loss of gene dosage [35]. Patient 2 is best described as having a contiguous gene deletion syndrome in which CTCF haploinsufficiency accounts for the neurodevelopmental component, but the contribution of neighboring genes to the non-neurological features cannot be formally partitioned in a single patient; attributing the entire presentation to CTCF alone would be inappropriate. We suggest three specific additions to the surveillance of Patient 2: a skeletal survey including clavicular radiographs and assessment of dentition, since clavicular hypoplasia and dental anomalies are consistent features of CLCD2 and have not yet been evaluated; periodic ophthalmological review given the codeleted HSF4; and the monitoring of blood pressure and serum potassium given the codeleted HSD11B2. A complete annotation of the deleted interval is provided in Supplementary Table S1.

Several limitations should be acknowledged. First, this was a retrospective study of four patients from a single center, and the small sample size precludes formal genotype–phenotype correlation analysis. Second, the diagnostic assays varied among patients and genome sequencing was not performed in any patient, so the possibility of additional pathogenic variants in regions not covered by the assays employed cannot be excluded. Third, the large 16q21-q22.1 deletion in Patient 2 encompasses 86 additional annotated loci, and the contribution of these codeleted genes cannot be determined from a single case. Fourth, the breakpoints of the exon 8–10 deletion in Patient 4 were not resolved, and no RNA-level or protein-level studies were performed to confirm transcript structure or CTCF abundance; the functional predictions for this variant therefore remain in silico inferences. Fifth, follow-up durations were variable and relatively short, precluding the assessment of late-onset features including the theoretical tumor risk associated with CTCF haploinsufficiency, for which current evidence does not support routine surveillance [36,37]. Finally, the association between a CTCF variant and congenital chylothorax rests on a single observation and requires independent replication, and the retrospective extraction of phenotypic data from clinical records may have introduced ascertainment bias.

## 5. Conclusions

We describe four unrelated Chinese children carrying four distinct classes of CTCF lesion: a zinc finger missense substitution, a frameshift truncating the protein upstream of both the cohesin-anchoring motif and the zinc finger array, an in-frame intragenic deletion of exons 8 to 10, and a whole-gene deletion within a large contiguous deletion. Although these variants differ in type, they converge on the loss of normal CTCF function, though by different routes. The whole-gene deletion and the frameshift reduce the amount of functional protein available at all binding sites. The missense substitution and the in-frame multi-exon deletion are predicted to yield a protein that is made but engages a restricted subset of CTCF sites. The phenotypic spectrum in our series ranged from complete developmental catch-up by 4 years of age to a critical neonatal multisystem presentation. These findings expand the mutational and phenotypic spectrum of CTCF-related disorder in the Chinese population and highlight the marked clinical heterogeneity of CTCF-related disorders. In children with unexplained developmental delay and multisystem involvement, a genome-wide approach rather than gene-targeted testing is recommended. In neonates presenting with unexplained chylothorax and multisystem abnormalities, especially when accompanied by features suggestive of a neurodevelopmental syndrome, CTCF should be considered in the differential diagnosis. Future studies incorporating transcriptomic and epigenomic analyses in patient-derived cells or model systems will be valuable for dissecting the allele-specific mechanisms underlying this heterogeneity.

## Figures and Tables

**Table 1:** The clinical characteristics of the four patients.

Characteristic	Patient 1	Patient 2	Patient 3	Patient 4
Sex	Male	Female	Female	Female
Age at presentation	3 years and 11 months	10 months	1 year	5 days (neonate)
Gestation	Full-term	Full-term	Full-term	Preterm (33w 3d)
Birth weight	3.4 kg	2.65 kg	3.0 kg	1.74 kg (AGA for prematurity)
Birth length	Not reported	48 cm	49 cm	46 cm (AGA for prematurity)
Weight (at last follow-up)	53 kg (>P97, age 8y 6mo)	7.8 kg (<P3, age 1y 10mo)	21 kg (P90–P97, age 4y 8mo)	10.71 kg (P75–P90, CA 11 mo 26 d)
Height (at last follow-up)	145 cm (>P97, age 8y 6mo)	78.1 cm (<P3, age 1y 10mo)	122 cm (>P97, age 4y 8mo)	76.8 cm (P75–P90, CA 11 mo 26 d)
Microcephaly	No	Yes (HC 41.4 cm at 10mo, <P3)	No	Yes (HC 38 cm at CA 4 mo 23 d, <P3)
Facial dysmorphism	Hypertelorism	Frontal bossing, hypertelorism, low-set ears, flattened nasal tip	Left-sided strabismus	Low-set ears
Global developmental delay	Yes (Gesell DQ: Adaptive 34, Gross Motor 47, Fine Motor 28, Language 33, Personal–Social 21)	Yes (Gesell DQ: Adaptive 69, Gross Motor 50, Fine Motor 64, Language 50, Personal–Social 69)	Yes (Gesell DQ: Adaptive 79, Gross Motor 96, Fine Motor 86, Language 67, Personal–Social 71)	No (Griffiths: All subdomains at 10–11 mo level at CA 11 mo 26 d)
Autistic-like behaviors	Yes	Yes	No	No
Brain anomalies (MRI/CT)	Imaging at 18 months: congenital brain developmental abnormalities, right hippocampal gliosis	Nodular isointense T2 lesions in the fourth ventricle; obstructive hydrocephalus; widened extra-axial spaces of bilateral temporal lobes	Cystic lesion in right temporal pole	Mildly decreased white matter density in bilateral frontoparietal lobes; dilated left lateral ventricle
Other significant findings	Single transverse palmar crease	Large anterior fontanelle	No	Neonatal chylothorax with severe multisystem disease

**Table 2:** Primers used for Sanger validation.

Primer Name	Primer Sequence		
*CTCF*-F	GCCTCCCAAGTAATGACCAA		
*CTCF*-R	CTGCTCTGCCCAGCTCTACT		
PCR Program			
Step	Temperature (°C)	Time	Cycles
Initial Denaturation	95	3–5 min	1
Denaturation	95	15–30 s	35
Annealing	55	15–30 s	
Extension	72	30 s	
Final Extension	72	5–10 min	
Hold	4	∞	

**Table 3:** A molecular and clinical summary of the four CTCF variants identified in this study.

Feature	Patient 1	Patient 2	Patient 3	Patient 4
Variant type	Missense	Whole-gene deletion (contiguous gene deletion)	Frameshift (4 bp deletion)	Intragenic multi-exon deletion
Genomic description (GRCh37)	NC_000016.9:g.67654630C>T	NC_000016.9:g.58986875_67907636del	NC_000016.9:g.67645349_67645352del	-
cDNA (NM_006565)	c.1117C>T	Whole gene deleted	c.615_618delGAAA	Deletion of exons 8–10
Protein (NP_006556.1)	p.(His373Tyr)	—	p.(Lys206Profs*15)	In-frame deletion; predicted loss of 160 codons (453–613) (LOVD reading frame checker)
Protein region affected	ZF4 (aa 351–373); His373 = second Zn-coordinating His	Entire CTCF protein; 86 further annotated loci codeleted	N-terminal domain, upstream of the YxF/CES cohesin-binding motif (aa 222–231)	Exons encoding the C-terminal part of ZF7 and the whole of ZF8-ZF11
Zygosity	Heterozygous	Heterozygous loss (x1)	Heterozygous	Heterozygous
Detection method	WES; CMA and MLPA negative	WES and CNV-seq	WES	Copy number analysis of WES data
Inheritance	De novo	De novo	De novo	De novo
Population frequency	Absent from ExAC, ESP6500 and 1000 Genomes; gnomAD	—	Absent from gnomAD v4.1.0	—
Previously reported	Yes—c.1117C>T recorded in ClinVar (VCV001308122.2); two further substitutions of the same codon (p.His373Asp, p.His373Pro) reported by Konrad et al. [18] → PM5	Region-level entity already recognized: 16q22 deletion syndrome (OMIM #614,541) [19]; CTCF deletion syndrome [20]	Yes—Chen et al. [17]; also listed by Konrad et al. [18]	Other intragenic CTCF deletions reported [18]; an exon 8–10 deletion not identified in the databases searched
ACMG/ClinGen classification	Likely pathogenic	Pathogenic	Pathogenic	Pathogenic
Evidence codes	PS2 + PM1 + PM5 + PM2_Supporting + PP3 [21]	Scored under the ACMG/ClinGen copy-number standard [22]: contains an established haploinsufficient gene (CTCF) + de novo occurrence	PVS1 + PS2 + +PM2_Supporting [21,23]	PVS1_Strong + PS2 + PM2_Supporting [21,23] (PVS1 downgraded from very strong: the deletion is in frame but removes a region critical to DNA binding)
Key clinical features	Global developmental delay; autism-like behaviors; overgrowth (>P97); hypertelorism; single transverse palmar crease	Severe pre- and postnatal growth restriction; microcephaly; enlarged anterior fontanelle; frontal bossing; global developmental delay; autism-like behaviors	Mild language and personal–social delay; strabismus; overgrowth (>P97); full catch-up by 4 years 8 months	Preterm (33 + 3 weeks); congenital chylothorax; respiratory and heart failure; PDA/PFO; hypotonia; mild global lag at CA 11 mo 26 d

## Data Availability

The individual-level clinical, laboratory, neuroimaging, and genetic sequencing data generated and analyzed during this study are not publicly available to protect the privacy of the pediatric participants. However, de-identified data that support the main findings of this study may be made available from the corresponding author upon reasonable request. Any data sharing will be subject to a formal data use agreement and require approval from the Ethics Committee of Beijing Children’s Hospital.

## Supplementary Materials

The following supporting information can be downloaded at: https://www.mdpi.com/article/10.3390/genes17090995/s1, Table S1: The annotation of all annotated loci within the 16q21-q22.1 deletion of Patient 2; Table S2: The diagnostic investigations performed in each of the four patients; Table S3: The database search record supporting the variant novelty statements; Figure S1: The output of the LOVD exonic deletion reading frame checker for the deletion of exons 8–10 of CTCF (NM_006565.3) in Patient 4.
