# Supplementary material for: A Genotype–Phenotype Analysis of Four Chinese Children Carrying Distinct Pathogenic Variants in the CTCF Gene
Source: Genes (Basel). 2026 Aug 24;17(9):995. doi: 10.3390/genes17090995 (PMC13606757; doi:10.3390/genes17090995)
Supplement: Supplementary file 1 [file genes-17-00995-s001.zip › Table S3. The database search record supporting the variant novelty statements.pdf]

Table S3. The database search record supporting the variant novelty statements

Resource	Version / release	Search term or interval	Date accessed	Result	Interpretation
**ClinVar**	current	Gene: CTCF; and chr16:58,986,875-67,907,636 (GRCh37) copy-number variants	August 2026	c.615_618delGAAA = VCV000521287;c.1117C>T =VCV001308122.2	One reported allele; c.1117C>T supported by PM5
**ClinGen Dosage Map**	current	HGNC:13723 (CTCF); each gene in the deleted interval	August 2026	CTCF haploinsufficiency score 3 (sufficient evidence)	Supports PVS1 and the copy-number classification
**DECIPHER**	current	chr16:58,986,875-67,907,636	August 2026	87 annotated loci retrieved	Basis of Table S1
**dbVar**	current	chr16:58,986,875-67,907,636 structural variants	August 2026	No overlapping report matching the present deletion	8.92-Mb deletion not previously reported at this size
**gnomAD**	v4.1.0	CTCF single-nucleotide variants and structural variants	August 2026	c.1117C>T and c.615_618delGAAA absent	Supports PM2_Supporting
**LOVD reading-frame checker**	current	CTCF, NM_006565.3, deletion of exons 8-10	August 2026	IN FRAME (exons 8-10 = c.1358_1837, 480 nt, a multiple of three)	PVS1 downgraded to PVS1_Strong for Patient 4 (in-frame deletion removing a region critical to DNA binding)
**OMIM**	current	*604167; #615502; #614541; #620099; #616553; #614937; #117210; #116800; #218030	August 2026	Entries reviewed for each dosage-relevant gene	Basis of the attribution column in Table S1
**PubMed**	n/a	(CTCF[gene]) AND (chylothorax OR "multisystem" OR neonat*), 2024-2026	August 2026	**no relevant literature reports**	
