# Supplementary material for: A Genotype–Phenotype Analysis of Four Chinese Children Carrying Distinct Pathogenic Variants in the CTCF Gene
Source: Genes (Basel). 2026 Aug 24;17(9):995. doi: 10.3390/genes17090995 (PMC13606757; doi:10.3390/genes17090995)
Supplement: Supplementary file 1 [file genes-17-00995-s001.zip › Figure S1. The output of the LOVD exonic deletion reading frame checker for the deletion of exons 8–10 of CTCF (NM_006565.3) in Patient 4.pdf]

Global Variome shared LOVD

https://databases.lovd.nl/shared/scripts/readingFrameChecker.php?gene=CTCF&transcript=NM_006565.3&variant=del&exon_from=8&exon_to=10

LOVD exonic deletions/duplications reading-frame checker

The predictions are based on direct translation of the mRNA, which is generated by deletion / insertion (duplication) of the exons as selected by the user. Please note that for data derived from analysis of DNA the result is just a prediction based on these data only; without confirmation on RNA level (experimental evidence), this prediction does not provide certainty and cannot be used as evidence for the effect which the change detected will have on RNA level. Literature reports several exceptions where changes at DNA level do not match exactly with changes on RNA level. For example, on RNA-level more exons might be missing because signals required for correct splicing are disrupted or deleted. In addition, intronic sequences flanked by inefficient splicing signals (so called 'cryptic' splice sites) might be activated yielding newly recognized exons incorporated in the mRNA.

Currently viewing gene/transcript: **CTCF / NM_006565.3**

Deletion or Duplication	Deletion
From exon	8
To exon	10
	Check

Deleting exon 8 to exon 10 leads to ... an IN-FRAME deletion.

According to the CTCF_NM_006565.3 reference sequence in the LOVD database, the HGVS notation of this deletion is:
ex08ex10del -> c.1358-?_1837+?del -> **c.(1357+1_1358-1)_(1837+1_1838-1)del**
