# Supplementary material for: A Genotype–Phenotype Analysis of Four Chinese Children Carrying Distinct Pathogenic Variants in the CTCF Gene
Source: Genes (Basel). 2026 Aug 24;17(9):995. doi: 10.3390/genes17090995 (PMC13606757; doi:10.3390/genes17090995)
Supplement: Supplementary file 1 [file genes-17-00995-s001.zip › Table S1. The annotation of all annotated loci within the 16q21-q22.1 deletion of Patient 2.pdf]

Table S1. The annotation of all annotated loci within the 16q21-q22.1 deletion of Patient 2 (chr16:58,986,875-67,907,636, GRCh37; 8.92 Mb)

Gene content retrieved from DECIPHER; OMIM morbid status and inheritance from OMIM; dosage sensitivity from the ClinGen Dosage Sensitivity Map; constraint metrics from gnomAD v4.1.0. Filtering: L1 = protein-coding; L2 = dosage-sensitive (ClinGen haploinsufficiency score 3 or 2, or gnomAD pLI >= 0.9 / LOEUF < 0.35); L3 = associated with a Mendelian phenotype in OMIM.

No.	Gene	Location	Biotype	OMIM gene	OMIM phenotype (MIM)	Inheritance	ClinGen HI	ClinGen TS	pLI	LOEUF	Filter L1/L2/L3	Feature in Patient 2 potentially related	Attribution
1	**ACD**	16:67657512-67660810	protein_coding	616553	616553	AD, AR	No Evidence	No Evidence	0	0.92	Pass	None observed	Unlikely. Dominant allele is an in-frame TEL-patch deletion (p.Lys170del) that abolishes telomerase recruitment; ClinGen haploinsufficiency = no
2	**AGRP**	16:67482571-67483548	protein_coding	602311	601665	AD, AR, Mu	-	-	0.02	1.09	Fail L3	None observed	Susceptibility locus for obesity, not a Mendelian gene.
3	**APOOP5**	16:59754461-59755026	transcribed_processed_pseudogene	-	-	-	-	-	-	-	Fail L1	None observed	No Mendelian phenotype in OMIM; no evidence for dosage sensitivity.
4	**ATP6V0D1**	16:67438005-67481188	protein_coding	607028	-	-	-	-	0.97	0.51	Pass	None observed	No Mendelian phenotype in OMIM; no evidence for dosage sensitivity.
5	**ATP6V0D1-DT**	16:67481314-67505977	lncRNA	-	-	-	-	-	-	-	Fail L1	None observed	No Mendelian phenotype in OMIM; no evidence for dosage sensitivity.
6	**B3GNT9**	16:67148104-67151260	protein_coding	-	-	-	-	-	0.02	1.94	Pass	None observed	No Mendelian phenotype in OMIM; no evidence for dosage sensitivity.
7	**BEAN1**	16:66427295-66493529	protein_coding	612051	117210	AD	-	-	0	1.45	Pass	None observed	Unlikely. SCA31 is caused by an intronic pentanucleotide repeat insertion acting through RNA gain of function, not by loss of dosage.
8	**BEAN1-AS1**	16:66469796-66481230	lncRNA	-	-	-	-	-	-	-	Fail L1	None observed	No Mendelian phenotype in OMIM; no evidence for dosage sensitivity.
9	**C16orf86**	16:67666814-67668757	protein_coding	-	-	-	-	-	0	1.43	Pass	None observed	No Mendelian phenotype in OMIM; no evidence for dosage sensitivity.
10	**CA7**	16:66844414-66854153	protein_coding	114770	-	-	-	-	0.88	0.57	Pass	None observed	No Mendelian phenotype in OMIM; no evidence for dosage sensitivity.
11	**CARMIL2**	16:67644988-67657569	protein_coding	610859	618131	AR	-	-	0	0.7	Pass	None observed	Carrier state only (autosomal recessive).
12	**CBFB**	16:67028984-67101058	protein_coding	121360	620099	AD	0 No Evidence	0 No Evidence	0.2	1.06	Pass	Enlarged anterior fontanelle (4 x 4 cm); frontal bossing; delayed dentition	Plausible contributor. Cleidocranial dysplasia 2 (MIM 620099), autosomal dominant, heterozygous loss-of-function mechanism established
13	**CDH11**	16:64943753-65126112	protein_coding	600023	211380	AR	-	-	1	0.25	Pass	None observed	Carrier state only (autosomal recessive).
15	**CDH16**	16:66908122-66919187	protein_coding	603118	-	-	-	-	0	1.08	Pass	None observed	No Mendelian phenotype in OMIM; no evidence for dosage sensitivity.
16	**CDH5**	16:66366636-66404784	protein_coding	601120	-	-	-	-	0	0.66	Fail L3	None observed	No Mendelian phenotype in OMIM; murine homozygous null is embryonic lethal. Retained in the table because of its role in endothelial/lymphatic
17	**CDH8**	16:61647242-62037035	protein_coding	603008	-	-	-	-	1	0.37	Fail L3	None observed	No OMIM phenotype; autism candidate gene. LoF-intolerant (pLI 1.00, LOEUF 0.37).
18	**CDH8-AS1**	16:61918321-61940836	lncRNA	-	-	-	-	-	-	-	Fail L1	None observed	No Mendelian phenotype in OMIM; no evidence for dosage sensitivity.
19	**CENPT**	16:67828157-67847811	protein_coding	611510	618702	AR	-	-	0	0.92	Pass	None observed	Carrier state only (autosomal recessive).
20	**CES2**	16:66934444-66945096	protein_coding	605278	-	-	-		0	1	Pass	None observed	No Mendelian phenotype in OMIM; no evidence for dosage sensitivity.
21	**CES3**	16:66961245-66975149	protein_coding	605279	-	-	-	-	0	0.99	Pass	None observed	No Mendelian phenotype in OMIM; no evidence for dosage sensitivity.
22	**CES4A**	16:66988585-67010417	protein_coding	621153	-	-	-	-	0	0.95	Pass	None observed	No Mendelian phenotype in OMIM; no evidence for dosage sensitivity.
23	**CIAO2B**	16:66932065-66934423	protein_coding	614778	-	-	-	-	0	1.68	Pass	None observed	No Mendelian phenotype in OMIM; no evidence for dosage sensitivity.
24	**CKLF**	16:66552563-66566251	protein_coding	616074	-	-	-	-	0	1.72	Pass	None observed	No Mendelian phenotype in OMIM; no evidence for dosage sensitivity.
25	**CKLF-CMTM1**	16:66552587-66579135	protein_coding		-	-	-	-	0	1.85	Pass	None observed	No Mendelian phenotype in OMIM; no evidence for dosage sensitivity.
26	**CMTM1**	16:66566393-66579137	protein_coding	607884	-	-	-	-	0.01	1.04	Pass	None observed	No Mendelian phenotype in OMIM; no evidence for dosage sensitivity.
27	**CMTM2**	16:66579448-66588275	protein_coding	607885	-	-	-	-	0	1.31	Pass	None observed	No Mendelian phenotype in OMIM; no evidence for dosage sensitivity.
28	**CMTM3**	16:66603874-66613892	protein_coding	607886	-	-	-	-	0.25	0.8	Pass	None observed	No Mendelian phenotype in OMIM; no evidence for dosage sensitivity.
29	**CMTM4**	16:66614750-66696743	protein_coding	607887	-	-	-	-	0.01	1.14	Pass	None observed	No Mendelian phenotype in OMIM; no evidence for dosage sensitivity.
30	**CTCF**	16:67561831-67639189	protein_coding	604167	615502	AD	3(Sufficient Evidence)	No Evidence	1	0.2	Pass	Global developmental delay; microcephaly; growth restriction	Principal driver. Only gene in the interval with an established autosomal dominant, haploinsufficiency-mediated neurodevelopmental phenotype
31	**CTCF-DT**	16:67490771-67563958	lncRNA	-	-	-	-	-	-	-	Fail L1	None observed	No Mendelian phenotype in OMIM; no evidence for dosage sensitivity.
32	**DPPA3P11**	16:62808222-62808706	processed_pseudogene	-	-	-	-	-	-	-	Fail L1	None observed	No Mendelian phenotype in OMIM; no evidence for dosage sensitivity.
33	**DUXAP11**	16:59655602-59656336	processed_pseudogene		-	-	-	-	-	-	Fail L1	None observed	No Mendelian phenotype in OMIM; no evidence for dosage sensitivity.
34	**DYNC1LI2**	16:66720893-66751836	protein_coding	611406	-	-	-	-	1	0.47	Pass	None observed	No Mendelian phenotype in OMIM; no evidence for dosage sensitivity.
35	**DYNC1LI2-DT**	16:66751752-66754740	lncRNA	-	-	-	-	-	-	-	Fail L1	None observed	No Mendelian phenotype in OMIM; no evidence for dosage sensitivity.
36	**E2F4**	16:67192155-67198918	protein_coding	600659	-	-	-		0.36	0.61	Pass	None observed	No Mendelian phenotype in OMIM; no evidence for dosage sensitivity.
37	**EDC4**	16:67873049-67884516	protein_coding	606030	-	-	-	-	0.59	0.49	Fail L1	n/a	Partially deleted — lies across the distal breakpoint.
38	**ELMO3**	16:67199111-67204029	protein_coding	606422	-	-	-		0	1.09	Pass	None observed	No Mendelian phenotype in OMIM; no evidence for dosage sensitivity.
39	**ENKD1**	16:67662945-67667265	protein_coding	621119	-	-	-	-	0	1.47	Pass	None observed	No Mendelian phenotype in OMIM; no evidence for dosage sensitivity.
40	**EXOC3L1**	16:67184314-67190242	protein_coding	614117	-	-	-	-	0	1.17	Pass	None observed	No Mendelian phenotype in OMIM; no evidence for dosage sensitivity.
41	**FBXL8**	16:67159932-67164570	protein_coding	609077	-	-	-	-	0	1.98	Pass	None observed	No Mendelian phenotype in OMIM; no evidence for dosage sensitivity.
42	**FBXL9P**	16:67207605-67226998, 16:67207139-67227048	lncRNA		-	-	-	-	-	-	Fail L1	None observed	No Mendelian phenotype in OMIM; no evidence for dosage sensitivity.
43	**FHOD1**	16:67229387-67247539	protein_coding	606881	-	-	-	-	0	0.92	Pass	None observed	No Mendelian phenotype in OMIM; no evidence for dosage sensitivity.
44	**GFOD2**	16:67674531-67719339	protein_coding	619933	-	-	-	-	0.74	0.61	Pass	None observed	No Mendelian phenotype in OMIM; no evidence for dosage sensitivity.
45	**GNPATP**	16:60654235-60656649	processed_pseudogene	-	-	-	-	-			Fail L1	None observed	No Mendelian phenotype in OMIM; no evidence for dosage sensitivity.
46	**HSD11B2**	16:67430652-67437553	protein_coding	614232	218030	AR	-	-	0	1.2	Pass	None observed (normotensive, normokalaemic)	Carrier state only (autosomal recessive). Blood pressure and serum potassium monitoring advised.
47	**HSF4**	16:67164681-67169945	protein_coding	602438	116800	AD	-	-	0	1.11	Pass	None observed (no cataract to date)	Uncertain. Autosomal dominant cataract (MIM 116800), but reported alleles are missense; consequence of whole-gene loss unknown.
48	**KCTD19**	16:67289428-67326760	protein_coding	619943	-	-	-	-	0	0.74	Fail L3	None observed	No human phenotype in OMIM (murine meiosis defect only).
49	**LINC00920**	16:66408504-66412135	lncRNA	-	-	-	-	-	-	-	Fail L1	None observed	No Mendelian phenotype in OMIM; no evidence for dosage sensitivity.
50	**LINC00922**	16:65284493-65576352	lncRNA	-	-	-	-	-	-	-	Fail L1	None observed	No Mendelian phenotype in OMIM; no evidence for dosage sensitivity.
51	**LINC02126**	16:65135990-65176795	lncRNA	-	-	-	-	-	-	-	Fail L1	None observed	No Mendelian phenotype in OMIM; no evidence for dosage sensitivity.
52	**LINC02141**	16:59855353-60053973	lncRNA	-	-	-	-	-	-	-	Fail L1	None observed	No Mendelian phenotype in OMIM; no evidence for dosage sensitivity.
53	**LINC02165**	16:63730170-63755884	lncRNA	-	-	-	-	-	-	-	Fail L1	None observed	No Mendelian phenotype in OMIM; no evidence for dosage sensitivity.
54	**LRRC36**	16:67326798-67385204	protein_coding	-	-	-	-	-	0	0.9	Pass	None observed	No Mendelian phenotype in OMIM; no evidence for dosage sensitivity.
55	**MATCAP1**	16:67175599-67184228	protein_coding	619978	-	-	-		0	0.97	Pass	None observed	No Mendelian phenotype in OMIM; no evidence for dosage sensitivity.
56	**MIR328**	16:67202321-67202395	miRNA	613701	-	-	-	-	-	-	Fail L1	None observed	No Mendelian phenotype in OMIM; no evidence for dosage sensitivity.
57	**NAE1**	16:66802874-66873256	protein_coding	603385	620210	AR	-	-	0	0.85	Pass	None observed	Carrier state only (autosomal recessive).
58	**NOL3**	16:67170154-67176189	protein_coding	605235	614937	AD	-	-	0	1.85	Pass	None observed	Unlikely. Single family, missense allele; Nol3-null mice have no motor phenotype; adult-onset phenotype.
59	**NPAP1L**	16:60358455-60359480	unprocessed_pseudogene	-	-	-	-	-			Fail L1	None observed	No Mendelian phenotype in OMIM; no evidence for dosage sensitivity.
60	**NUTF2**	16:67846867-67872569	protein_coding	605813	-	-	-	-	0.99	0.4	Pass	None observed	No Mendelian phenotype in OMIM; no evidence for dosage sensitivity.
61	**PARD6A**	16:67660909-67662777	protein_coding	607484	-	-	-	-	-	-	Pass	None observed	No Mendelian phenotype in OMIM; no evidence for dosage sensitivity.
62	**PDP2**	16:66878589-66895754	protein_coding	615499	-	-	-	-	0	0.96	Pass	None observed	No Mendelian phenotype in OMIM; no evidence for dosage sensitivity.
63	**PHAF1**	16:67109941-67148544	protein_coding	-	-	-	-	-	0.2	0.6	Pass	None observed	No Mendelian phenotype in OMIM; no evidence for dosage sensitivity.
64	**PLEKHG4**	16:67277510-67289499	protein_coding	609526	-	-	-	-	0	0.72	Pass	None observed	No Mendelian phenotype in OMIM; no evidence for dosage sensitivity.
65	**PPIAP48**	16:64736794-64737283	transcribed_processed_pseudogene	-	-	-	-	-	-	-	Fail L1	None observed	No Mendelian phenotype in OMIM; no evidence for dosage sensitivity.
66	**RANBP10**	16:67723070-67806560	protein_coding	614031	-	-	-	-	0.99	0.48	Pass	None observed	No Mendelian phenotype in OMIM; no evidence for dosage sensitivity.
67	**RIPOR1**	16:67518418-67546788	protein_coding	619842	-	-	-	-	0	0.61	Pass	None observed	No Mendelian phenotype in OMIM; no evidence for dosage sensitivity.
68	**RN7SKP118**	16:67321658-67321987	misc_RNA	-	-	-	-	-	-	-	Fail L1	None observed	No Mendelian phenotype in OMIM; no evidence for dosage sensitivity.
69	**RN7SKP76**	16:61743154-61743476	misc_RNA	-	-	-	-	-	-	-	Fail L1	None observed	No Mendelian phenotype in OMIM; no evidence for dosage sensitivity.
70	**RN7SL543P**	16:67019519-67019817	misc_RNA	-	-	-	-	-	-	-	Fail L1	None observed	No Mendelian phenotype in OMIM; no evidence for dosage sensitivity.
71	**RNA5SP428**	16:66301809-66301940	rRNA_pseudogene	-	-	-	-	-	-	-	Fail L1	None observed	No Mendelian phenotype in OMIM; no evidence for dosage sensitivity.
72	**RNU1-123P**	16:67390691-67390850	snRNA	-	-	-	-	-	-	-	Fail L1	None observed	No Mendelian phenotype in OMIM; no evidence for dosage sensitivity.
73	**RNU4-58P**	16:59540126-59540266	snRNA	-	-	-	-	-	-	-	Fail L1	None observed	No Mendelian phenotype in OMIM; no evidence for dosage sensitivity.
74	**RNU6-21P**	16:62187540-62187646	snRNA	-	-	-	-	-	-	-	Fail L1	None observed	No Mendelian phenotype in OMIM; no evidence for dosage sensitivity.
75	**RPS15AP34**	16:63350763-63351149	processed_pseudogene	-	-	-	-		-	-	Fail L1	None observed	No Mendelian phenotype in OMIM; no evidence for dosage sensitivity.
76	**RPS27AP16**	16:61055445-61055914	transcribed_processed_pseudogene	-	-	-	-	-	-	-	Fail L1	None observed	No Mendelian phenotype in OMIM; no evidence for dosage sensitivity.
77	**RPS27P27**	16:59088837-59089091	processed_pseudogene	-	-	-	-		-	-	Fail L1	None observed	No Mendelian phenotype in OMIM; no evidence for dosage sensitivity.
78	**RRAD**	16:66921682-66926308	protein_coding	179503	-	-	-	-	0	1.12	Pass	None observed	No Mendelian phenotype in OMIM; no evidence for dosage sensitivity.
79	**SLC9A5**	16:67237683-67272191	protein_coding	600477	-	-	-	-	0	0.72	Pass	None observed	No Mendelian phenotype in OMIM; no evidence for dosage sensitivity.
80	**TERB1**	16:66754640-66801620	protein_coding	617332	619646	AR	-	-	0	1.07	Pass	None observed	Carrier state only (autosomal recessive).
81	**THAP11**	16:67842320-67844195	protein_coding	609119	620940	AR	-	-	0.52	0.68	Pass	None observed	Carrier state only (autosomal recessive).
83	**TK2**	16:66508003-66552544	protein_coding	188250	617069	AR	-	-	0	1.1	Pass	None observed	Carrier state only (autosomal recessive).
85	**TMEM208**	16:67227103-67229278	protein_coding	620781	-	-	-	-	0	1.02	Pass	None observed	No Mendelian phenotype in OMIM; no evidence for dosage sensitivity.
86	**TPPP3**	16:67389027-67393518	protein_coding	616957	-	-	-	-	0.08	0.95	Pass	None observed	No Mendelian phenotype in OMIM; no evidence for dosage sensitivity.
87	**TRADD**	16:67153969-67163760	protein_coding	603500	-	-	-	-	0	0.89	Pass	None observed	No Mendelian phenotype in OMIM; no evidence for dosage sensitivity.
88	**TSNAXIP1**	16:67806765-67832148	processed_pseudogene	607720	-	-	-	-	0	1.23	Fail L1	None observed	No Mendelian phenotype in OMIM; no evidence for dosage sensitivity.
89	**UBE2FP2**	16:63180610-63181071	processed_pseudogene	-	-	-	-	-	-	-	Fail L1	None observed	No Mendelian phenotype in OMIM; no evidence for dosage sensitivity.
90	**ZDHHC1**	16:67394152-67416833	protein_coding	-	-	-	-	-	0	1.29	Pass	None observed	No Mendelian phenotype in OMIM; no evidence for dosage sensitivity.

Total: 87 annotated loci, of which 57 are protein-coding. CTCF is the only gene in the interval with an established autosomal dominant, haploinsufficiency-mediated neurodevelopmental phenotype.
