# Supplementary material for: A Genotype–Phenotype Analysis of Four Chinese Children Carrying Distinct Pathogenic Variants in the CTCF Gene
Source: Genes (Basel). 2026 Aug 24;17(9):995. doi: 10.3390/genes17090995 (PMC13606757; doi:10.3390/genes17090995)
Supplement: Supplementary file 1 [file genes-17-00995-s001.zip › Table S2. The diagnostic investigations performed in each of the four patients.pdf]

Table S2. The diagnostic investigations performed in each of the four patients

	Patient 1	Patient 2	Patient 3	Patient 4
**Karyotype**	Not performed	Not performed	Not performed	Not performed
**Chromosomal microarray (CytoOneArray)**	Performed – normal	Not performed	Not performed	Not performed
**MLPA (SALSA P070, P106)**	Performed – normal	Not performed	Not performed	Not performed
**Whole-exome sequencing**	Performed	Performed	Performed	Performed
**Copy-number variation sequencing**	Not performed	Performed	Not performed	Not performed
**Exome-based copy-number analysis**	Performed – normal	Performed	Performed	Performed
**Whole-genome sequencing**	Not performed	Not performed	Not performed	Not performed
**Sanger validation (parent–offspring trio)**	Performed – de novo	Performed – de novo	Performed – de novo	Performed – de novo
**Assay that detected the reported variant**	Whole-exome sequencing	Copy-number variation sequencing	Whole-exome sequencing	Exome-based copy-number analysis
**Other clinically relevant findings**	None	None	None	None

Genomic breakpoints of the exon 8-10 deletion in Patient 4 could not be resolved from exome coverage data and were not reported by the referring laboratory. The deletion spans c.1358_1837 (480 nt) and is in frame (LOVD reading-frame checker, NM_006565.3; Figure S1).
